# Peritonsilar abscess requiring intensive care unit admission caused by group C and G *Streptococcus*: a case report

**DOI:** 10.4076/1757-1626-2-6808

**Published:** 2009-09-11

**Authors:** Nidhi Gupta, Justin Lovvorn, Robert M Centor

**Affiliations:** Department of General Internal Medicine, University of Alabama at Birmingham(1530 3rd Ave. South), Birmingham, (35294-3407)USA

## Abstract

Acute adult pharyngitis is a common reason to visit the primary care physician’s office. Without knowledge of the natural course of acute pharyngitis in the adult patient, it can be easy to miss a serious complication. We offer the case of a 46 year-old man who initially presented with acute pharyngitis and eventually developed a peritonsillar abscess requiring intubation and intensive care unit admission. We hope to further clarify the normal natural history of adult pharyngitis and suggest clinical guidelines in the event of worsening pharyngitis.

## Introduction

Acute pharyngitis represents one of the ten most frequent reasons for episodic care. Rarely, pharyngitis can progress to serious complications. Group A beta-hemolytic streptococcus is only one of several bacterial pharyngitis etiologies. Research has shown that group C beta-hemolytic streptococcus is also an important source in adolescents and young adults [1]. We report a man who had culture proven group C and group G pharyngitis, yet tested positive on the rapid antigen tests for group A pharyngitis. His symptoms worsened until he developed a peritonsillar abscess with edema and marked airway occlusion. We suggest clinical guidelines in the event of worsening pharyngitis. Furthermore, this case should remind primary care physicians of the natural history of pharyngitis and its suppurative complications.

## Case presentation

A 46-year-old white male presented to the emergency department (ED) complaining of progressive dyspnea. He was healthy until three days prior to hospital admission when he developed a sore throat and fever. He went to his primary care physician’s (PCP) office where he had a positive group A rapid antigen test. His PCP gave him a prescription for cefdinir 300 mg twice daily for ten days and he began taking the antibiotic. His throat felt worse the next day and his fever increased to 105 degrees Fahrenheit. He called the PCP’s office but was not given an office appointment that day. On day 3, he returned to the PCP’s office with complaints of persistent fever, sore throat and new onset of redness and swelling over the left side of his neck. He received a shot of ceftriaxone and promethazine (to treat his nausea) and was told to put an ice pack on his neck swelling. That night, the patient’s wife noted he began acting confused and had some trouble sleeping. The following morning, the patient developed severe shortness of breath with intermittent air gasping. His wife called EMS, who transported him emergently to the hospital. The ED physicians observed him in acute respiratory distress leading to intubation. In addition to sedation/paralytic meds for intubation, he was given acetaminophen 650 mg per rectum, clindamycin 600 mg IV, ceftriaxone 2 gm IV, vancomycin 1 gm IV, and two liters of normal saline bolus. In the ED, he was later noted to have falling systolic blood pressures to the 60’s and was started on a dopamine drip and admitted to the ICU.

His past medical history included impaired fasting glucose and hypercholesterolemia, without history of surgeries. His home medications consisted of metformin 500 mg twice daily, resuvastatin 5 mg nightly, fish oil, flax seed, and niacin. Socially, he currently resides with his wife, denies tobacco or drug use and drinks alcohol occasionally.

On initial physical exam, his temperature was 104 degrees Fahrenheit. His first recorded blood pressure and heart rate were 141/81 and 117 respectively, but systolic blood pressure dropped to the 60s after some time in the ED. After intubation, his respiratory rate was 20 and his oxygen saturation was 100% on the ventilator.

In general, he was an average sized white male who seemed comfortable on the ventilator. He had normal sclera, conjunctiva and mucous membranes, but his oropharynx was poorly visualized due to ET and OG tubes in place. A large firm swelling over his left neck, which was erythematous and warm, was noted. His lungs were clear to auscultation bilaterally without wheezes or crackles and his cardiac exam revealed a fast rate and regular rhythm with S1 and S2 audible, without murmurs, rubs, or gallops. His peripheral pulses were strong and no significant edema was noted. His abdomen was soft and nondistended with positive bowel sounds. His skin had no evident rashes or lesions. The neurological exam and mental status was poorly tested due to paralytics given in the ED.

Initial laboratory data revealed a white blood cell count of 6.96, hemoglobin of 13.2, hematocrit of 38.4, and platelet count of 134. The basic metabolic panel showed sodium of 125, potassium of 4.0, chloride of 91, bicarbonate of 22, BUN of 7, creatinine of 0.9, glucose of 140, and calcium of 8.5. The hepatic panel was within normal limits. His cardiac enzymes, including CK, CKMB and troponin, were normal. Initial arterial blood gas after intubation showed a pH of 7.13, pCO2 of 61, pO2 of 136, and oxygen saturation of 98% on 100% O2. After ventilator adjustment the pH normalized to 7.41 with a pCO2 of 35.

**Figure 1.**
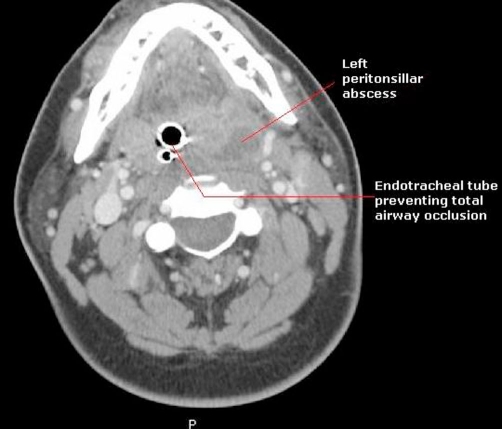
Computer tomography (CT) scan of neck. Left peritonsillar abscess causing airway occlusion. Total airway occlusion is prevented by an endotracheal tube.

On imaging, his head computerized tomography (CT) scan showed no acute disease. His neck CT scan showed extensive soft tissue edema along the nasopharynx and orophyarnx involving the prevertebral and parapharyngeal spaces, in addition to a left peritonsillar abscess that completely obliterated the airspace except for the endotracheal tube (Figure 1).

**Figure 2.**
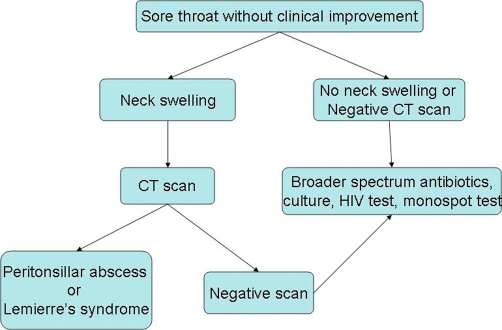
Recommended guidelines for further evaluation of worsening adult pharyngitis.

The patient was started on dexamethasone intravenously soon after admission. The otolaryngologist performed a bedside incision and drainage (I&D) of the abscess. Over the next couple of days, the patient was weaned off the ventilator and dopamine was discontinued. Due to an adverse reaction to clindamycin on day 1 of hospitalization, we changed his antibiotic regimen to vancomycin and piperacillin-tazobactam. On hospital day 4, we extubated the patient. Unfortunately, that night he had a recurrence of dyspnea with stridor, which required transfer back to the ICU with repeat I&D of the abscess. After the repeat drainage, the patient had a smooth recovery and was discharged 3 days later on continued antibiotics and oral steroids. His final peritonsillar wound culture results revealed group C and group G streptococcus and his blood cultures indicated septicemia with group C streptococcus.

## Discussion

Group A *streptococci* are the major microbial pathogen causing endemic adult pharyngitis, but studies have shown that group C *streptococci* is also an important pathogen in adult pharyngitis. Current pharyngitis guidelines focus only on group A streptococci and only recommend antibiotics for this type of pharyngitis. It is important for the primary care physician to consider the other possibilities in a patient with pharyngitis, especially when that patient is not following the natural course of group A streptococcal pharyngitis.

This case report illustrates the importance of understanding the natural history of the suspected disease process for the primary care physician. According to the Centor criteria, streptococcal pharyngitis is a likely diagnosis when the patient presents with three out of four of the following: fever, absence of cough, tonsillar exudates and tender cervical lymphadenopathy [2]. Treatment for streptococcal pharyngitis is utilized to prevent suppurative complications, prevent rheumatic fever, improve clinical signs and symptoms, and reduce transmission [3]. Even without treatment, streptococcal pharyngitis should improve in five to seven days. With treatment, the patient should be showing clinical improvement in three days. If the patient is not improving within three days of antibiotics, it is necessary to consider further diagnoses. It is easy to miss an unusual cause of adult pharyngitis given that current guidelines focus only on group A *streptococcus*. We suggest the following guidelines in a case of adult pharyngitis that is either worsening or not improving within three days of antibiotics. If the patient has neck swelling, it is imperative to perform a CT scan of the neck. This will help rule out peritonsillar abscess or Lemierre’s syndrome. It is important to note that peritonsillar abscess can develop even in the setting of antibiotic therapy. If the patient has no neck swelling or a negative CT scan, then consider throat culture, broader spectrum antibiotics, and HIV testing along with a monospot test (Figure 1).

As indicated above, in the face of worsening pharyngitis, the differential must be broadened to include group C and G streptococcal pharyngitis, peritonsillar abscess, acute HIV, infectious mononucleosis, and in some cases Lemierre’s syndrome. These conditions usually present with a negative rapid antigen detection test. Although our patient had a positive rapid antigen detection test (discussed below), it is still necessary to have an extensive differential due to our patient’s deteriorating condition.

Group C and group G streptococcal pharyngitis are important causes of pharyngitis and should be included in the differential of a patient with worsening pharyngitis. Group C streptococcal pharyngitis has a prevalence of less than 5% in adult patients, but is significant to differentiate because it can also lead to rising ASO titers, glomerulonephritis, and pharyngitis epidemics^1^. Perhaps more importantly, group C *streptococcus* infection can result in extreme suppurative complications as demonstrated by our patient. There are some differences between group A and group C streptococcal pharyngitis. For example, patients with group C streptococcal pharyngitis can have shorter fever, lower oral temperatures, and less tonsillar enlargement and exudates compared to group A streptococcal pharyngitis [1]. However, despite minor differences, group A and group C and G streptococcal pharyngitis can often be clinically indistinguishable [4,5]. Therefore, the wary clinician should consider group C and group G streptococcal pharyngitis in a patient presenting with sore throat. Studies indicate that penicillin is still a good antibiotic choice for group C and G pharyngitis, along with cephalosporins and vancomycin [6]. However, group G streptococcus potentially has a higher rate of resistance to vancomycin [7].

Group C and G streptococcal infection might have been easier to determine in our patient if the rapid antigen detection test (RADT) was negative. The RADT is designed to detect group A beta-hemolytic streptococcus and has a sensitivity between 86-94% depending on the type of RADT being utilized [8]. It is quite unusual that our patient had a positive rapid antigen detection test when his cultures did not grow group A streptococcus. It is atypical for the RADT to have a false positive, but a commensal bacterium called *Streptococcus milleri* and variants of group A streptococci can both produce a positive RADT. Another possibility is that our patient was also initially infected with Group A streptococcus, which was not detected on culture due to antibiotic treatment. On the other hand, group C and G streptococcus grew in culture because they were present in the peritonsillar abscess. Generally, group C and group G streptococci will be negative on RADT due to their lack of a group A carbohydrate antigen [8]. An exhaustive literature search did not reveal any relationship between group C and group G streptococcal pharyngitis and a positive RADT.

Our patient’s diagnosis should have been suspected by his unilateral neck swelling. Peritonsillar abscess was confirmed by CT scan of the neck and culture was taken during incision and drainage. In the United States, there are approximately 45,000 cases of peritonsillar abscess per year. Despite the abundance of cases, treatment varies greatly among otolaryngologists including initial choice of antibiotics. According to Hanna et al., beta hemolytic streptococci play a large role in peritonsillar abscess. Group A streptococcus was the most common isolate, followed by bacteroides and then group C streptococcus [9]. The bacterial isolates present in peritonsillar abscess are important due to the high number of anaerobic bacteria.

Empiric treatment with broad spectrum antibiotics has been suggested in peritonsillar abscesses due to the prominence of anaerobes in culture isolates. However, it is likely that penicillin is still the best option following incision and drainage or needle aspiration, even if the isolates contain anaerobes. This is due to the fact that most anaerobes are still sensitive to penicillin [10-13]. An exception to this rule is the bacteroides species, but patients with that particular infection recovered with the addition of metronidazole [10]. Kieff et al. found that following incision and drainage of the abscess, an antibiotic treatment regimen with penicillin is equal to a regimen with broad spectrum antibiotics [14]. This suggests that when dealing with peritonsillar abscess the first step is to drain the abscess, followed by an antibiotic regimen with penicillin with progression to a broader spectrum antibiotic if the patient does not show improvement.

Adequate usage of antibiotics for sore throat has been shown to decrease incidence of peritonsillar abscess. A review conducted by the Cochrane Collaboration revealed that treating sore throat with antibiotics reduced peritonsillar abscess versus treatment with placebo. Most of the studies reviewed used penicillin as their antibiotic of choice. However, one study used sulfonamides and one used either penicillin or cefixime [3]. As emphasized earlier, it is important to remember that peritonsillar abscess can occur even though the patient had adequate antibiotic treatment.

Bacterial infections play a large role in pharyngitis, but there are important viral causes to be considered as well. Infectious mononucleosis caused by the Epstein-Barr virus (EBV) is another pathogen to consider in a patient with worsening pharyngitis. EBV is most commonly observed among young adults and is spread through the saliva. EBV often resembles streptococcal pharyngitis due to the thick exudates and palatal petechiae but, unlike streptococcal pharyngitis, has posterior and anterior cervical tender lymphadenopathy. EBV infection can be complicated by peritonsillar abscess and up to 1.6% of patients with peritonsillar abscess have positive heterophile antibody production. Treatment by abscess tonsillectomy was found to be safe in patients with infectious mononucleosis [15]. Our patient did not have risk factors for EBV and improved with incision and drainage along with antibiotics; therefore, he did not require a heterophile antibody test.

Human immunodeficiency virus (HIV) is another viral infection to consider in a patient that presents in a similar fashion to our patient. Pharyngitis can present in patients as the initial manifestation of HIV as a part of the acute retroviral syndrome. HIV pharyngitis often manifests acutely and resembles a mononucleosis-like syndrome. This form of pharyngitis usually has tonsillar hypertrophy without exudates. HIV pharyngitis has not been associated with peritonsillar abscess or other suppurative complications. However, it is important to remember in a patient with sore throat due to the gravity of the diagnosis. Our patient did not have the proper risk factors or presentation for HIV pharyngitis therefore was not tested.

Finally, Lemierre’s syndrome is a rare but potentially life threatening condition that cannot be ignored, particularly in the setting of pharyngitis with neck swelling. Lemierre’s syndrome is an acute oropharyngeal infection with secondary thrombophlebitis of the internal jugular vein caused by the bacteria *Fusobacterium necrophorum*. Lemierre’s syndrome can be complicated by septic emboli, especially to the brain or lungs. Unfortunately, it is most often found in young and healthy patients who have higher morbidity if hospitalization is delayed [16]. These patients often have high fevers (39-41°C) and rigors, which occur four to five days after the onset of a sore throat. Other features can include cough, pleuritic chest pain, and pleural effusions. Signs of metastatic abscesses include nodular lesions in the chest, empyema, septic arthritis, and soft tissue abscesses [17]. Despite the rarity of the disease, the incidence of Lemierre’s syndrome is increasing for unknown reasons [18]. Therefore, it is important to test for this condition in the case of worsening pharyngitis and neck swelling with a CT scan in order to prevent delay of hospitalization. Our patient is not the correct age for Lemierre’s syndrome and the CT scan did not show infiltration of the internal jugular vein.

Diagnosing adult pharyngitis can become complex, especially when the patient does not follow the normal natural history of the disease. Our case reminds us of the importance of understanding the natural history of streptococcal pharyngitis and our suggested guidelines provide a model on how to treat those patients that fall outside of those parameters. If more primary care physicians follow these guidelines, we can quickly diagnose early complications of adult pharyngitis.

## Patient perspective

Day 1: I felt ill at work and checked out to go to the doctor. I had a sore throat and the doctor diagnosed me with strep throat and a sinus infection. I was given a ten day course of antibiotics.

Day 2: I continued to feel ill and my temperature went up to 105 degrees Fahrenheit. I called the doctor and they told me to stay on the antibiotics. I was a bit disappointed that my fever was not taken more seriously.

Day 3: My neck started to swell on the left side. I also had a high fever and some nausea. I had reflux with everything I drank. I went to see the doctor and got a shot of Rocephin and some pills for nausea. I felt that the doctor should have been more concerned, especially since I had a two inch painfully swollen mass on my left neck. I was told to treat the swelling with an ice pack.

Day 4: I was hoping to feel better but instead I woke up with extreme difficulty breathing. I got out of bed to go to the bathroom, which is about twenty five feet. Even this short walk was too much. I made it to the sink before I had to stop and control my breathing. My breaths were loud, shallow, and painful. I was not able to get enough air and I began to panic. While I was standing there gasping for air, I lost control of my bowel and bladder. I continued to stand there for a while trying to catch my breath. Then I stripped off my clothes, went to the toilet, and tried to clean myself. Still gasping for air, I moved as fast as I could through the house looking for my wife. I found her outside and desperately banged on the window and then on the front door. I was still breathing poorly and was anxious, but I felt more in control. I went to get dressed and my wife came running in the house. We agreed to call 911. The last thing I remember is getting dressed.

Days 5-6: I do not recall anything from these days but I was told later that I was sedated and intubated and the abscess was drained. I was also told I had very low blood pressure. Luckily, my two sisters, who are nurses, were here to help us understand what had happened.

Day 7: I woke up in the ICU and thought it had only been one day! I was feeling much better and the doctors transferred me out of the ICU to the floor. Things got worse overnight.

Day 8: I started getting nauseated around two o’clock am. I was given a pill for nausea but still slept little that night. I could feel the swelling in my tonsil come back and I choked on a pill around five o’clock am. I tried to take some extra water to help with the pill but I started to panic again. It seemed like twenty people came into the room. My breathing was very loud, shallow, and painful. Again, I urinated a little and passed some gas. I was embarrassed by the noise I was making but had no control over it. The doctors tried to keep me calm and I tried to calm myself as well, but I felt as if I had no control over my own body. I was given oxygen and some breathing treatments, which seemed to help. They put in a transfer to the ICU. This took a couple hours and in that time I tried to relax and pray. In the ICU the ENT doctor drained the abscess and we continued to suction it for the next 24 hours. Draining the abscess, along with heavy steroids, seemed to work wonders and I improved over the next 36 hours.

Day 9: This was the first good day in a while. I was sent back to the floor at the end of the day and was finally able to get up and take a shower.

Day 10: The only problem I really had left was some mild shortness of breath and a low resting pulse, but there were no abnormalities from the workup. I was able to go home after lunch. I was very happy with my hospital stay.

Course at home: I tried to get back into shape so I could go back to work. I tried to walk and sit up for extended periods of time. I still had some drainage which gave me a sore throat. I also had some diarrhea. Overall, I lost 15 pounds in about two weeks.

## Abbreviations

CT: computerized tomography
EBV: Epstein-Barr virus
ED: emergency department
HIV: Human Immunodeficiency Virus
I&D: incision and drainage
PCP: primary care physician
RADT: rapid antigen detection test
